# Editorial: The next stage of immune cell design: selective targeting of multi-antigen profiles

**DOI:** 10.3389/fimmu.2025.1585904

**Published:** 2025-03-17

**Authors:** Alexander Kamb, Jackson G. Egen

**Affiliations:** ^1^ Discovery Research, A2 Biotherapeutics, Agoura Hills, CA, United States; ^2^ Oncology Research, Gilead Sciences, Foster City, CA, United States

**Keywords:** multi-specific, logic gate, NOT gate, AND gate, OR gate, immunotherapy, immuno-oncology

The human genome sequence has been known in its entirety for nearly 25 years and genome-wide analysis underpins an unprecedented level of insight into the structure and function of human genes and their role in disease pathology. This comprehensive dataset, along with the technologies for decoding and interpreting it, has fueled many medical innovations, including new diagnostics and treatments for disease. However, such comprehensive understanding—down to the quantum unit of genetics, the nucleotide—has raised concerns that the field is approaching “genome exhaustion,” an asymptote in the discovery of monogenic causes of disease. The rate of discovery of simple, large-effect mechanisms that can be therapeutically exploited is falling because the genome has been scoured with such powerful methods. In addition, many drivers of disease remain undruggable with current approaches, limiting the near-term therapeutic potential of these mechanistic findings. Thus, new approaches are urgently needed for the untreated causes and manifestations of disease.

**Figure 1:**
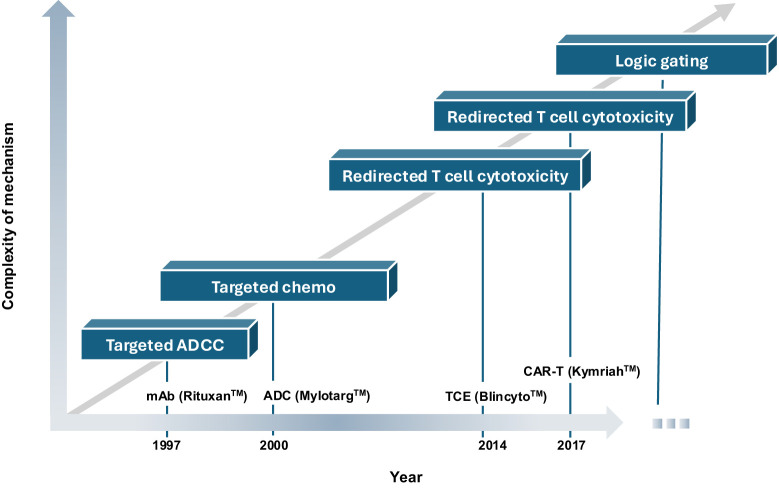
Historical timeline of progress toward logic-gated therapeutics. ADCC, antibody-dependent cellular cytotoxicity; mAb, monoclonal antibody; ADC, antibody-drug conjugate; TCE, T-cell engager; CART, chimeric antigen-receptor T cell.

Multi-specific engineered proteins and cell therapies are exciting, innovative approaches that exploit existing knowledge of biological pathways and molecular targets to treat disease more effectively. Enabled by technological advances in protein and cell engineering and manufacturing, these modalities form the basis of therapeutics of greater complexity to address grievous illness, without necessarily depending on the discovery of new genes (**Figure 1**). Instead, they enable endogenous or exogenous immune cells to recognize the multi-antigen profiles of pathogenic cells, driving efficient elimination while sparing healthy tissues.

In this Research Topic, state-of-the-art multi-specific designs are discussed in a series of informative papers. Biological processes require specificity and cells and multi-specific proteins are uniquely equipped to execute complex targeted behaviors. One well-understood example involves the immune response to infection, where the multi-functional properties of antibodies and the effector activity of T cells help to orchestrate the selective destruction of pathogens and pathogen-infected cells. These mechanisms have been co-opted in oncology to create cell- and protein-based medicines designed to target individual molecules expressed on cancer cells. Building on this foundation, research in drug discovery has advanced to the stage where systems for integrating signals derived from multiple antigens have been tested. This strategy broadens the prospect of cell and multi-specific-protein therapy, enabling successful application of these approaches across a large number of diseases and patient subsets with substantially reduced toxicity.

By analogy with binary computing devices, cell- and protein-based therapeutics designed to recognize multiple antigens are sometimes classified as types of synthetic logic, where responses are gated by the specific profile of multi-signal inputs. Such elements include cells engineered to integrate signals from two antigens in various ways: (i) OR gates where either of the two antigens activates a response; (ii) AND gates where both antigens must be present to activate; and (iii) NOT gates where one antigen must be present, and the second antigen absent, to activate. More complex signal integration is also possible. These concepts can be extended to protein therapeutics where the infused medicine comprises only the ligand-binding portion of receptors. The effector functions are supplied by the patient’s own immune cells, orchestrated by the multi-specific agent.

The series of articles begins with a historical account by Paul and Zhou of the key milestones in the development of antibodies, the modality which first made the linking together of functional modules feasible. Antibodies come naturally equipped with multiple modules for binding and effector function, and these can be augmented by adding warheads; e.g., toxins to treat cancer in more targeted ways.

To exploit antigen profiles, it is essential not only to understand expression patterns across cell types but also exploit them. Nix et al. describe the latest gene-expression technology that can be harnessed to find antigen profiles that distinguish one cell type from another, including tumor and normal cells. Nolan-Stevaux and Smith survey the variety of technologies, both protein- and cell-based, that can be utilized to “read” profiles on cancer cells. They compare the strengths and weaknesses of T-cell engagers and CAR-T cells that can execute simple logical operations to control immune-cell activation based on antigen inputs.

The final set of papers delves into focused examples of multi-specific designs. Dixon et al. describe a novel therapeutic approach where NK cells are first derived from iPSCs and then engineered with additional effector-targeting capabilities to provide a flexible platform that can distinguish cancer cells from healthy tissues. Argueta et al. build on a different effector cell in the immune system, the myeloid cell, and illustrate how *in vivo* delivery of synthetic mRNA to these cells holds promise for more convenient and cheaper multi-targeted therapies. Finally, DiAndreth et al. report a specific example of multi-receptor targeting by engineered T cells that use a NOT logic gate. In the case detailed here, the NOT gate interprets the absence of a given antigen as a signal to activate and kill AML cells specifically.

This Research Topic connects the first steps toward multi-specific targeting of therapeutics to the future of these biological modalities that is unfolding now in preclinical and clinical studies.

